# Mathematical Analysis of Pseudoplastic Polymers during Reverse Roll-Coating

**DOI:** 10.3390/polym12102285

**Published:** 2020-10-06

**Authors:** Fateh Ali, Yanren Hou, Muhammad Zahid, Muhammad Afzal Rana

**Affiliations:** 1 School of Mathematics & Statistics, Xi’an Jiaotong University, Xi’an 710049, China; 2 Department of Mathematics, COMSATS University Islamabad, Abbottabad Campus, Abbottabad 22060, Pakistan; zahid315@hotmail.com; 3 Department of Mathematics & Statistics, Riphah International University, Sector I-14, Islamabad 44000, Pakistan; mafzalrana@gmail.com

**Keywords:** non-Newtonian fluid, roll-coating analysis, lubrication approximation theory (LAT), perturbation technique, approximate solution, numeric methods

## Abstract

This article presents a mathematical model and theoretical analysis of coating of a thin film of non-Newtonian polymers as they travel through a small space between two reverse-rotating rolls. The dimensionless forms of the governing equations are simplified with the help of the lubrication approximation theory (LAT). By using the perturbation technique, the analytical solutions for velocity, flow rate and pressure gradient were obtained. From an engineering point of view, some significant results such as thickness of the coated web, pressure distribution, separation points, separation force and power input were computed numerically. The effect of velocities ratio k and Weissenberg number We on these physical quantities is presented graphically; others are shown in tabular form. It is noted that the involved material parameters provide a mechanism to control the flow rate, pressure distribution, the thickness of coating, separation force and power input. Moreover, the separation point is shifted toward the nip region by increasing velocities ratio k.

## 1. Introduction

Roll coating is an engineering procedure in which a uniform thin liquid film is deposited onto a substrate. The phenomenon of roll coating has gained a solid reputation in recent times due to its wide applications in coating industries. In many industrial processes, thin uniform liquid coatings are produced on surfaces with coating materials. Such procedures involve wrapping, wallpaper and adhesive tapes, beautification, books and magazine, plastic films, protection of fabrics or metals, X-ray films, photographic films, coated items, foils and coated paper and magnetic records, etc. These activities rely on a wide range of apparatuses, among which roll coaters are popular. In the roll-coating process, in which the radii of the rolls are much greater than nip distance between the two rotating rolls, when a fluid flows through such a small gap, it comes out as a thin liquid film that can be used to coat a surface. The flow of fluid in a small space between a pair of rotating rollers is the key factor controlling the thickness and uniformity of the coated film. The coating thickness depends primarily on the gap between the adjacent rollers and their relative speeds. According to the direction of the rolls, roll coating is classified as reverse roll-coating (RRC), metering roll-coating and forward roll-coating [1,2,3,4]. In the case of forward roll-coating, the two rolls at the nip go in the same direction. The coating fluid forms a bath on the upstream side of the nip and, after leaving the nip, divides into two liquid films that are transferred onto both sides of the roller—one of them is applied for industrial purposes to a web. In reverse roll-coating, the rollers move in the opposite direction and the metering coating on the nip [5,6,7].

A pioneering work including experimental, analytical and computational analysis related to the roll-coating process, can be found in [5,8,9]. In an earlier work, Coyle et al. [10] used a finite element approach backed by experimental outcomes to explain the essential fluid dynamic characteristics of the reverse roll-coating. They also clarified the existence of flow instabilities, including ribbing and cascading. Taylor and Zettlemoyer et al. [11] used the lubrication theory to perceive ink flow behavior in the process of printing press. They obtained the influences of pressure distribution and force. The water flow between two rolls was discussed by Hinter Maier and White et al. [12]. They used the principle of lubrication and verified the findings which were compatible with their experimental results. Greener and Middleman et al. [13] and Ho and Holland et al. [14] developed mathematical models based on the lubrication theory to examine reverse roll-coating systems; however, they ignored the influences of surface tension, existence of free surfaces and dynamics of contact lines. Jang et al. [6] used the finite-volume method and volume of fluid (VoF)-free surface technique to 3D model of non-Newtonian flow in reverse roll-coating. This related work was carried out on inelastic non-Newtonian fluids. The power-law exponent was considered in the range of 0.95 to 1.05. Mainly concentration was paid on the resulting thickness of the coating and the effect of roll-speed on ribbing instabilities. The results show that with the increase of the power-law index, the thickness of the coating film increases. Furthermore, considering dynamic of wetting lines, Shiode et al. [15] used VoF to analyze reverse roll-coating numerically. Their results reveal that the wetting line approaches the nip as the speed ratio increases. Recently, Zahid et al. [16] gave numerical results for second-grade materials by utilizing the lubrication approximation theory. Engineering parameters such as the thickness of the coating, pressure distribution, split location, strength, roll power consumption, stresses and adiabatic temperature rise was achieved among coating roll and coated web. Zahid et al. [17] discussed the Rabinowitz fluid numerically. They presented physical parameters of engineering interest such as stresses, force, separation position, pressure distribution, power input, temperature to rolls and thickness of the coating of the web.

Today, the occurrence of non-Newtonian fluids in mechanical, aeronautical and industrial engineering is more important than Newtonian materials. Because of this reason many non-Newtonian fluid models with different constitutive equations have been proposed [18,19,20,21,22]. The analysis of pseudoplastic fluids has gained importance due to its main uses in the industry [23]. Numerous non-Newtonian polymers are pseudoplastic and are being extensively studied. Pseudoplastic liquids can be established in essential applications such as extruded polymer films, emulsion-coated films such as polymer solutions, photographic films with a high molecular weight and melts. However, less consideration has been paid to the Williamson fluid model, which describes the properties of pseudoplastic fluids [24,25]. Nadeem et al. have debated the peristaltic flow of a Williamson fluid [26]. Cramer et al. [27] demonstrated with experimental evidence that this model is better suited to polymer solutions and particle suspensions than other models.

In this article, we provide a theoretical analysis for the reverse roll-coating process of Williamson fluid films, which is drawn through a small gap between two rolls rotating in opposite directions. With the help of the lubrication approximation theory, the governing equations are simplified. The analytical solutions for flow rate, velocity and pressure gradient are provided by using the perturbation technique. From an engineering point of view, some significant results like pressure distribution, thickness of coated web, separation points, separation force and power input are presented. It was found that the involved material parameters provide a device to control the flow rate, the coating thickness separation points, separation force and power input. Our results are presented both graphically and in tabular form. The following sections describe the mathematical formulation and analytical solution of the problem. At the end, results and discussion and conclusions are presented.

## 2. Mathematical Formulation

**Figure 1:**
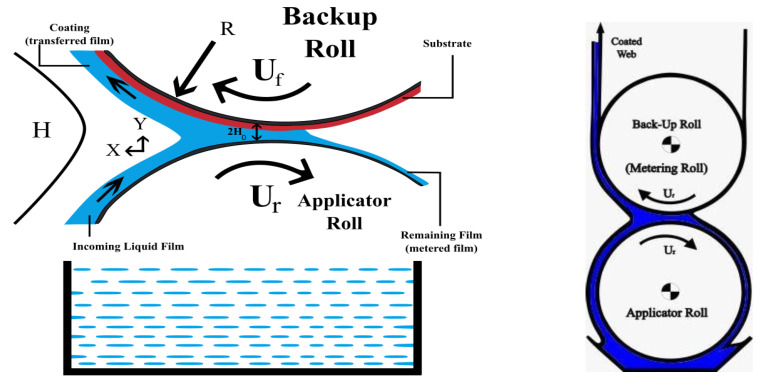
Geometry of reverse roll-coating (RRC).

We considered two dimensional steady, laminar flow of an incompressible Williamson fluid between two rolls rotating in the opposite direction with velocities $U_{f}=R\omega_{f}$ $U_{r}=R\omega_{r}$, where R is the radius of each roll and subscripts f and r stand for forward and reverse rotating rolls, respectively. The velocities ratio $k=\frac{U_{r}}{U_{f}}$ of both rolls is uniform, and the gap between rolls is $2H_{0}$. Moreover, the x−axis and y−axis are taken along and transversal to the flow direction, respectively, as represented in Figure 1.

The governing equations for the Williamson fluid are:

(1) $$\nabla .\bar{U}=0$$

(2) $$\rho \frac{D\bar{U}}{Dt}=-\nabla \bar{p}+\nabla .\bar{\tau }$$

where $\frac{D}{Dt}(*)=\frac{\partial }{\partial t}(*)+\bar{U}\cdot \nabla (*)$ denotes the material derivative, ρ presents the density, $\bar{p}$ is the pressure, $\bar{U}$ is the velocity and the extra stress tensor $\bar{\tau }$ for a Williamson fluid [28] is defined as:

(3) $$\bar{\tau }=\left[\mu_{\infty }+\left(\mu_{0}-\mu_{\infty }\right){\left(1-\Gamma \gamma \right)}^{-1}\right]{\bar{A}}_{1}$$

Here $\mu_{\infty }$ and $\mu_{0}$ denote the infinite and zero shear rate viscosities, respectively, ${\bar{A}}_{1}=\nabla \bar{U}+{\left(\nabla \bar{U}\right)}^{t}$ denotes the first Rivlin–Erickson tensor, and the time constant is denoted by Γ. The shear rate γ [26] is defined as:

(4) $$\gamma =\sqrt{\frac{1}{2}\sum_{ij}\sum_{ji}\gamma_{ij}\gamma_{ji}}=\sqrt{\frac{1}{2}\pi }$$

where $\pi =trace\left({\bar{A}}_{1}{}^{2}\right)$ represents the second tensor of invariant strain. When $\eta_{\infty }=0\;$ and  Γγ<1, then Equation (3) can be written as:

(5) $$\bar{\tau }=\mu_{0}(1+\Gamma \gamma ){\bar{A}}_{1}$$

The above model reduces to Newtonian for Γ=0. The two-dimensional velocity profile is taken as:

(6) $$\bar{U}=\left[\bar{u}(x,y),\bar{v}(x,y)\right]$$

In view of the Equation (6), the component forms of the governing Equations (1) and (2) can be written as:

(7) $$\frac{\partial \bar{u}}{\partial \bar{x}}+\frac{\partial \bar{v}}{\partial \bar{y}}=0$$

(8) $$\rho \left(\bar{u}\frac{\partial \bar{u}}{\partial \bar{x}}+\bar{v}\frac{\partial \bar{u}}{\partial \bar{y}}\right)=\frac{\partial {\bar{\tau }}_{xy}}{\partial \bar{y}}+\frac{\partial {\bar{\tau }}_{xx}}{\partial \bar{x}}-\frac{\partial \bar{p}}{\partial \bar{x}}$$

(9) $$\rho \left(\bar{u}\frac{\partial \bar{v}}{\partial \bar{x}}+\bar{v}\frac{\partial \bar{v}}{\partial \bar{y}}\right)=\frac{\partial {\bar{\tau }}_{yx}}{\partial \bar{y}}+\frac{\partial {\bar{\tau }}_{yy}}{\partial \bar{x}}-\frac{\partial \bar{p}}{\partial \bar{y}}$$

where ${\bar{\tau }}_{xx},\;{\bar{\tau }}_{yy},\;{\bar{\tau }}_{yx}$ and ${\bar{\tau }}_{xy}$ are the stress components.

## 3. The Dimensionless Form

Introducing the non-dimensional variables:

(10) $$\begin{array}{l} u^{*}=\frac{\bar{u}}{U_{f}},\;v^{*}=\frac{\bar{v}}{\delta U_{f}},\;x^{*}=\frac{\bar{x}}{{\left(RH_{0}\right)}^{\frac{1}{2}}},\;y^{*}=\frac{\bar{y}}{H_{0}}\\ p^{*}=\frac{\bar{p}H_{0}}{\mu_{0}U_{f}}{\left(\frac{H_{0}}{R}\right)}^{\frac{1}{2}},\;\gamma^{{}^{*}}=\frac{\gamma H_{0}}{U_{f}}\;,\;\delta =\sqrt{\frac{H_{0}}{R}} \end{array}\}$$

and dropping the steric (∗) for convenience, the dimensionless forms of the Equations (7)–(9) become:

(11) $$\frac{\partial u}{\partial x}+\frac{\partial v}{\partial y}=0$$

(12) Reδ(u∂u∂x+v∂u∂y)=δ∂τxx∂x+∂τxy∂y−∂p∂x

(13) Reδ3(u∂v∂x+v∂v∂y)=δ∂τyy∂y+δ2∂τxy∂y−∂p∂y

The stresses components involved in Equations (12) and (13) are given by:

(14) $$\tau_{yy}=2\delta \left[1+We\gamma \right]\frac{\partial v}{\partial y},\;\tau_{xx}=2\delta \left[1+We\gamma \right]\frac{\partial u}{\partial x},\;\tau_{xy}=\left[1+We\gamma \right]\left(\frac{\partial u}{\partial y}+\delta^{2}\frac{\partial v}{\partial x}\right)$$

where Re=UρH0μ0, $We=\frac{\Gamma U}{H_{0}}$ and

(15) $$\gamma ={\left[2\delta^{2}{\left(\frac{\partial v}{\partial y}\right)}^{2}+{\left(\frac{\partial u}{\partial y}+\delta^{2}\frac{\partial v}{\partial x}\right)}^{2}+2\delta^{2}{\left(\frac{\partial u}{\partial x}\right)}^{2}\right]}^{\frac{1}{2}}$$

Since δ is a square root of the ratio of $H_{0}$ to R and is very small, therefore ignoring the term having δ to get:

(16) $$\frac{\partial }{\partial y}\left\{\frac{\partial u}{\partial y}\left[We\frac{\partial u}{\partial y}+1\right]\right\}=\frac{\partial p}{\partial x}$$

(17) $$\frac{\partial p}{\partial y}=0$$

It is obvious from Equation (17) that p is not a function of y, so it is the function of x only, i.e., p=p(x). Thus, the Equation (16) becomes:

(18) $$\frac{\partial^{2}u}{\partial y^{2}}+We\frac{\partial }{\partial y}\left[{\left(\frac{\partial u}{\partial y}\right)}^{2}\right]=\frac{dp}{dx}$$

The dimensionless boundary conditions subjected to the Equation (18) are given by:

(19) $$\begin{array}{l} u=1\;at\;y=-\sigma \\ u=-k\;at\;y=\sigma \end{array}\}$$

where $\sigma =1+\frac{x^{2}}{2}$.

## 4. Solution of the Problem

The Equation (18) is a nonlinear differential equation; we will employ a regular perturbation technique for We<<1 to obtain its analytic solution. Therefore:

(20) $$u=u_{0}+Weu_{1}+We^{2}u_{2}\ldots ,$$

(21) $$\frac{dp}{dx}=\frac{dp_{0}}{dx}+We\frac{dp_{1}}{dx}+We^{2}\frac{dp_{2}}{dx}\ldots ,$$

(22) $$\lambda =\lambda_{0}+We\lambda_{1}+We^{2}\lambda_{2}\ldots ,$$

where $u_{0}$, $\frac{dp_{0}}{dx}$ and $\lambda_{0}$ are the zeroth-order solutions, which represent the Newtonian case [13], while $u_{1}$, $\frac{dp_{1}}{dx}$, $\lambda_{1}$, $u_{2}$, $\frac{dp_{2}}{dx}$ and $\lambda_{2}$ are the corrections up to first-order and second-order terms, respectively and have the contribution of non-Newtonian effects. By substituting Equations (20) and (21) into Equation (18) and comparing the same power of We, we obtain a system of differential equations as follows:

For $We^{0}$:

(23) $$\frac{d^{2}u_{0}}{dy^{2}}=\frac{dp_{0}}{dx}$$

with boundary conditions:

(24) $$\begin{array}{l} u_{0}=1\;at\;y=-\sigma \\ u_{0}=-k\;at\;y=\sigma \end{array}$$

For $We^{1}$:

(25) $$\frac{d^{2}u_{1}}{dy^{2}}+\frac{d}{dy}{\left(\frac{du_{0}}{dy}\right)}^{2}=\frac{dp_{1}}{dx}$$

with boundary condition:

(26) $$\begin{array}{l} u_{1}=0\;at\;y=-\sigma \\ u_{1}=0\;at\;y=\sigma \end{array}$$

### 4.1. Zeroth-Order Solution

The solution of zeroth-order boundary value problem (23) and (24) is given by

(27) $$u_{0}=\frac{1}{2}\left(y^{2}-\sigma^{2}\right)\frac{dp_{0}}{dx}-\frac{1}{2\sigma }\left(k+1\right)y+\frac{1}{2}\left(1-k\right)$$

Now, the zeroth-order dimensionless flow rate is defined as

(28) $$\lambda_{0}=\frac{1}{2}\int_{-\sigma }^{\sigma }u_{0}\left(y\right)dy$$

In addition, from Equations (27) and (28), we get

(29) $$\frac{dp_{0}}{dx}=-\frac{3\left(\left(k-1\right)\sigma +2\lambda_{0}\right)}{2\sigma^{3}}$$

The integration of Equation (29) with the condition $p_{0}=0$ as x→−∞ yields the zeroth-order pressure given by:

(30) $$p_{0}\left(x\right)=\frac{1}{16{\left(x^{2}+2\right)}^{2}}\left(\begin{array}{l} -18\sqrt{2}\left(\lambda_{0}+\frac{2}{3}\left(k-1\right)\right){\left(x^{2}+2\right)}^{2}\arctan\left(\frac{x\sqrt{2}}{2}\right)-9\pi \sqrt{2}\left(\lambda_{0}+\frac{2}{3}\left(k-1\right)\right){\left(x^{2}+2\right)}^{2}-\\ 36\left(\left(\lambda_{0}+\frac{2}{3}\left(k-1\right)\right)x^{2}+\frac{10}{3}\lambda_{0}+\frac{4}{3}\left(k-1\right)\right)x \end{array}\right)$$

To determine the thickness of the coating and the pressure distribution, we need to find the value of $\lambda_{0}\left(k\right)$. To meet this situation, the Swift–Stieber boundary condition on pressure is applied. It is claimed that at the transition point $x=x_{t}$ where a lubrication-type flow gives way to a transverse flow, both the pressure gradient and the pressure disappear. Upon setting $\frac{dp_{0}}{dx}=0$ in the Equation (29), we get:

(31) $$\sigma_{t}=1+\frac{x_{t}^{2}}{2}=\frac{2\lambda_{0}}{1-k}$$

Similarly, because of the Swift–Stieber boundary condition on pressure, replacing x with $x_{t}$ in Equation (30), finding value of $x_{t}$ in terms of $\lambda_{0}$ from the Equation (31) and substituting this value into the resulting equation from Equation (30), the transcendental equation in $\lambda_{0}$ is obtained. To handle the complexity of this equation for finding the valve of $\lambda_{0}$, we use the numeric technique, namely a regular false position method with a predefined accuracy of $10^{-10}$ and setting different valves of k.

### 4.2. First-Order Solution

The first-order solution procedure resembles to the zeroth-order solution. Using the Equation (23) into Equation (27) and employing boundary conditions (26) to obtain:

(32) $$u_{1}=-\frac{3\left(y+\sigma \right)\left(y-\sigma \right)\left(-\frac{\sigma^{6}}{6}\frac{dp_{1}}{dx}+\frac{\sigma^{3}}{4}\left(k^{2}-1\right)+\sigma^{2}\left(\frac{y}{4}{\left(k-1\right)}^{2}+\frac{k+1}{2}\lambda_{0}\right)+\sigma \left(k-1\right)y\lambda_{0}+{\left(\lambda_{0}\right)}^{2}y\right)}{\sigma^{6}}$$

The first-order dimensionless flow rate is defined as:

(33) $$\lambda_{1}=\frac{1}{2}\int_{-\sigma }^{\sigma }u_{1}\left(y\right)dy$$

From Equations (30) and (31), we have:

(34) $$\frac{dp_{1}}{dx}=\frac{3\left(k^{2}\sigma +2\lambda_{0}\left(k+1\right)-2\lambda_{1}\sigma -\sigma \right)}{2\sigma^{4}}$$

The first-order pressure is obtained by integrating Equation (34) with the condition $p_{1}=0$ as x→−∞ we get:

(35) $$p_{1}\left(x\right)=\frac{1}{32{\left(x^{2}+2\right)}^{3}}\left(\begin{array}{l} 30\sqrt{2}{\left(x^{2}+2\right)}^{3}\left(\left(k+1\right)\lambda_{0}+\frac{3}{5}\left(k^{2}-2\lambda_{1}-1\right)\right)\arctan\left(\frac{\sqrt{2}}{2}x\right)\\ +15{\left(x^{2}+2\right)}^{3}\left(\left(k+1\right)\lambda_{0}+\frac{3}{5}\left(k^{2}-2\lambda_{1}-1\right)\right)\pi \sqrt{2}\\ +60x\left(\begin{array}{l} \left(\left(k+1\right)\lambda_{0}+\frac{3}{5}\left(k^{2}-2\lambda_{1}-1\right)\right)x^{4}\\ +\left(\frac{16}{3}\left(k+1\right)\lambda_{0}+\frac{3}{5}\left(k^{2}-2\lambda_{1}-1\right)\right)x^{2}+\frac{44}{5}\left(k+1\right)\lambda_{0}+4k^{2}-8\lambda_{1}-4 \end{array}\right) \end{array}\right)$$

To obtain the value for $\lambda_{1}\left(k\right)$, the Swift–Stieber boundary condition on pressure will also be applied. It is claimed that the transition point $x=x_{t}$ where a lubrication-type flow provides a way to a transverse flow, both the pressure gradient and the pressure disappears. Upon setting $\frac{dp_{1}}{dx}=0$ in Equation (34), we get:

(36) $$\sigma =1+\frac{x_{t}^{2}}{2}=\frac{2\lambda_{0}\left(k+1\right)}{\lambda_{1}-3k^{2}+1}$$

Similarly, because of the Swift–Stieber boundary condition on pressure, replacing x with $x_{t}$ in Equation (35), finding the value of $x_{t}$ in terms of $\lambda_{1}$ from the Equation (36) and substituting this value into the resulting equation from Equation (35), transcendental equation in $\lambda_{1}$ is obtained. To handle the complexity of this equation for finding the valve of $\lambda_{1}$, we use numeric technique, namely a regular false position method with a predefined accuracy of $10^{-10}$ for different valves of k. These results are shown in Table 1 and Table 2.

Second simple material balance relation for λ can be written as:

(37) $$U_{f}H_{f}-U_{r}H_{r}=2\lambda H_{0}U_{f}$$

which may lead to the form:

(38) $$\upsilon =\frac{H_{f}}{H_{r}}=k+2ϒ\lambda$$

where $\upsilon =\frac{H_{f}}{H_{r}}$ is the thickness of the coating, $H_{r}$ and $H_{f}$ are the thickness of fluid films on the reverse roll and forward roll, respectively. The ratio $ϒ=\frac{H_{0}}{H_{r}}$ denotes half of the nip region $H_{0}$ to incoming fluid film $H_{r}$ from the reverse roll. Thus, we see that to find the thickness of the coating, it is necessary to know λ(k).

Thus, the perturbation solution up to first order is given by:

(39) $$\begin{array}{l} u=u_{0}+Weu_{1}\\ \lambda =\lambda_{0}+We\lambda_{1}\\ \frac{dp}{dx}=\frac{dp_{0}}{dx}+We\frac{dp_{1}}{dx} \end{array}\}$$

where $u_{0},\;u_{1},\;\frac{dp_{0}}{dx},\;\frac{dp_{1}}{dx}$ are given in Equations (27), (29), (32) and (34), respectively.

## 5. Operating Variables

When pressure distribution, velocity profile and pressure gradient are achieved, it is simple to find the operating variables such as separating force, power input, etc.

### 5.1. Separating Force

The separating force F in dimensionless form is given by:

(40) $$F=\frac{\bar{F}H_{0}}{\mu_{0}URW}=\int_{-\infty }^{x_{t}}p\left(x\right)dx$$

where $\bar{F}$ is the non-dimensional separating force per unit width W.

### 5.2. Power Input

The power transferred by the roll to the fluid is obtained by the integral:

(41) $$p_{w}=\frac{\bar{P}}{\mu_{0}WU^{2}}=\int_{-\infty }^{x_{t}}\tau \left(x,1\right)dx$$

where $P_{w}$ denotes the non-dimensional power. The non-dimensional shear stress is

(42) $$\tau_{xy}=\left(1+We\frac{\partial u}{\partial y}\right)\frac{\partial u}{\partial y}$$

## 6. Results and Discussion

**Figure 2:**
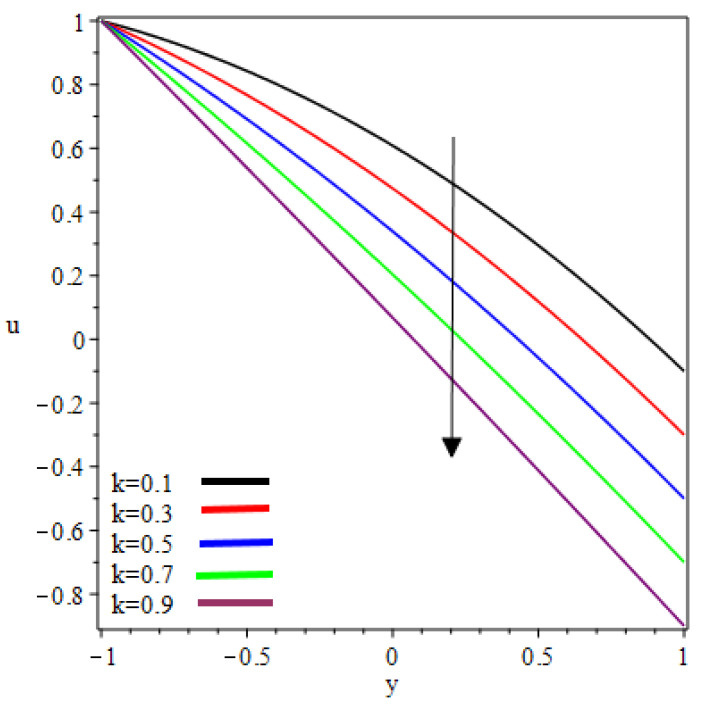
Influence on velocity profile at x=0.

**Figure 3:**
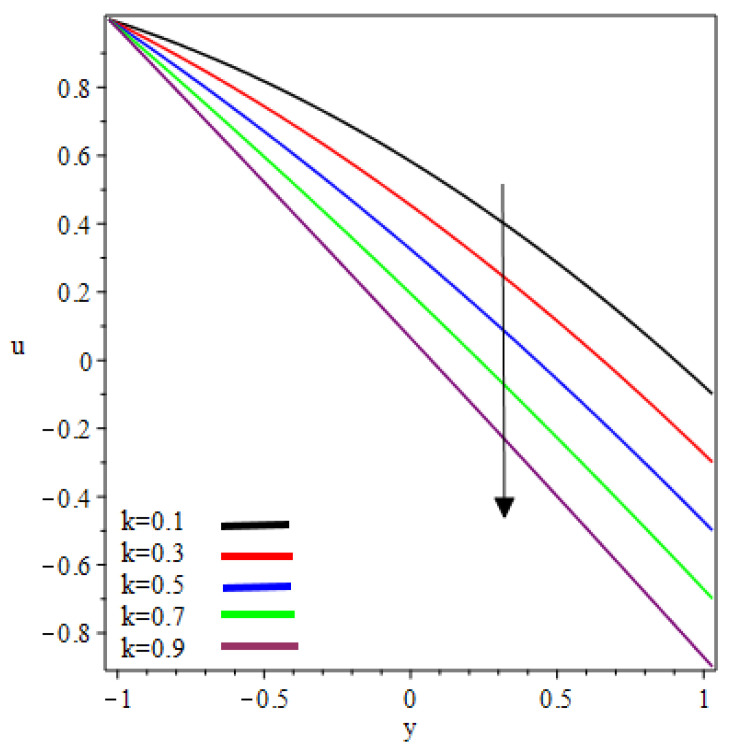
Influence on velocity profile at x=0.25.

**Figure 4:**
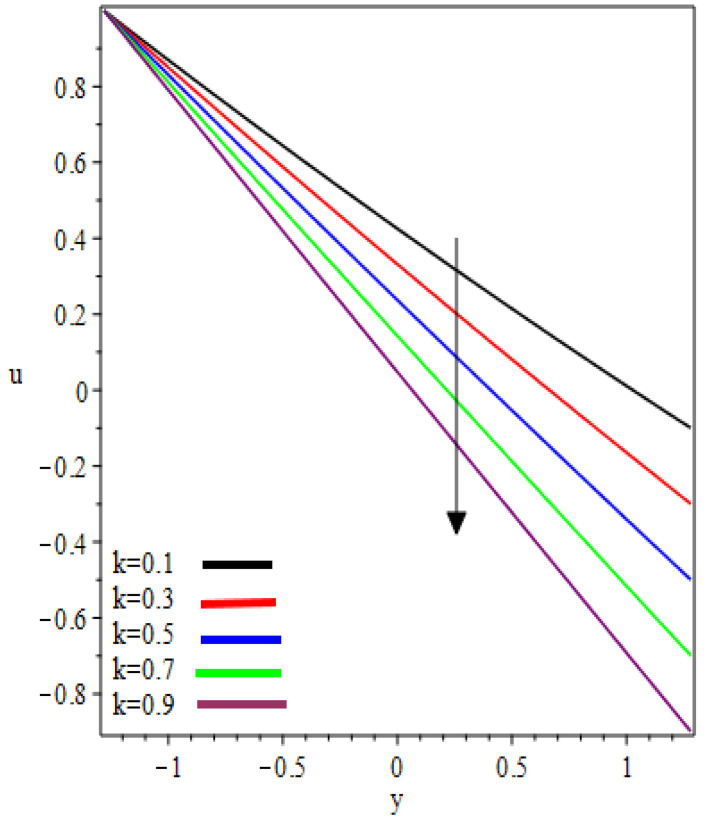
Influence on velocity profile at x=0.75.

**Figure 5:**
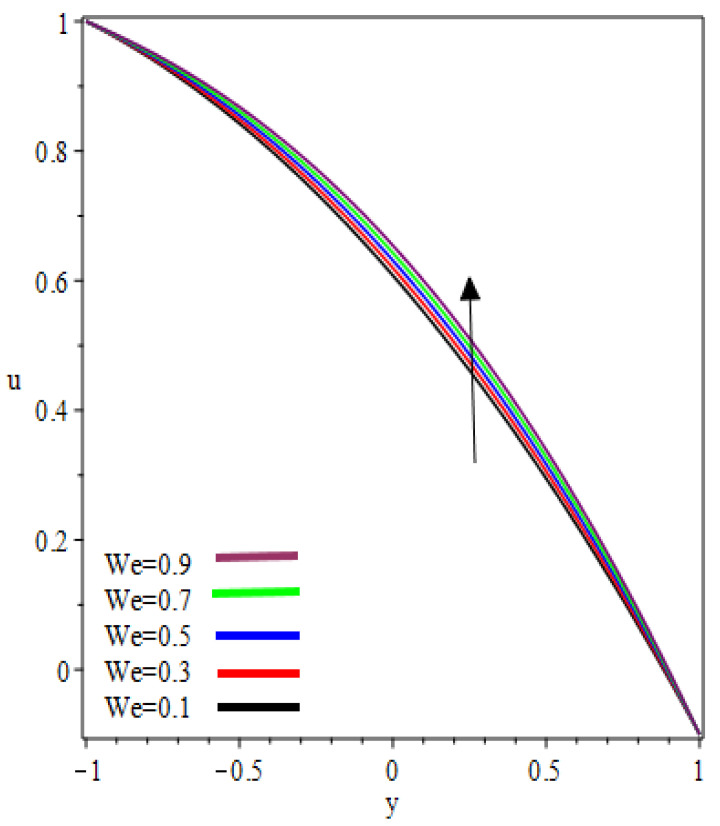
Influence on velocity profile at x=0.

**Figure 6:**
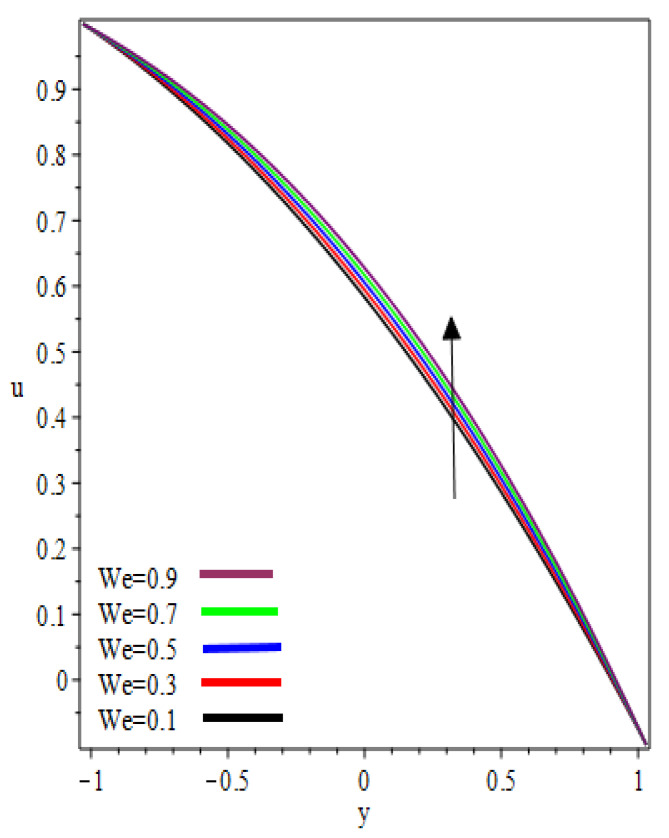
Influence on velocity profile at x=0.25.

**Figure 7:**
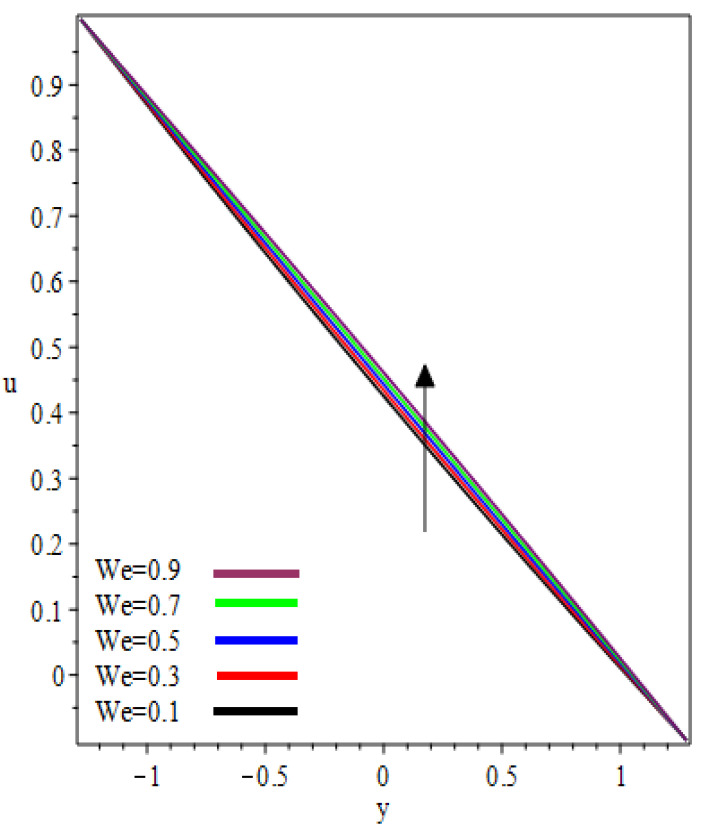
Influence on velocity profile at x=0.75.

This study examined the reverse roll-coating of an incompressible pseudoplastic material (Williamson fluid). The lubrication approximation theory was applied to simplify the governing equations of flow. The numeric results for flow rate λ, separation points $x_{t}$, thickness of the coating $\upsilon =\frac{H_{f}}{H_{r}}$, separation force F and power input $p_{w}$ transmitted to the roll are presented in Table 1 and Table 2 for several values of velocities ratio (the ratio of the velocity of the reverse roll to forward roll) k and Weissenberg number We. Figure 2, Figure 3, Figure 4, Figure 5, Figure 6 and Figure 7 present the non-dimensionless velocities outcomes at different positions x (0,0.25,0.75) in the reverse roll-coating process for the different values of involved material parameters k and We. The velocity in Figure 2 is sketched at the nip against the increasing value of k from 0.1 to 0.9. It was observed that the velocity profile decreases by increasing velocities ratio k. The maximum speed was found at the roll surface of the reverse roll. Then it starts decreasing while moving towards the forward roll and becomes zero when y∈[0.005,0.872], beyond this domain—depending upon the valve of k—one can see the reverse flow in the direction of the coating web. From Figure 3 and Figure 4, it is clear that, while moving toward the separation points at the different position of the reverse roll-coating process, the domain for y, where the velocity becomes zero is increasing. Beyond this domain, depending upon the ratio of velocities k while moving towards the upper roll, the magnitude of the velocity increases and attain its maximum speed at the surface of the roll. It is interesting to note that compatibility with the predictions of the model is quite suitable for small k, of course, deviations increases as k becomes large compared to unity. It is worth mentioning that the data for elastic polymer solutions agree well with the Newtonian theory [13] under the identical physical conditions. Figure 5, Figure 6 and Figure 7 indicate the velocity representations for the different values of We at various positions in the roll-coating process. These figures show that the velocity of the fluid increases by increasing We. Physically it means that the viscous forces are dominant over the elastic forces (because Weissenberg number is the ratio of viscous forces to elastic forces). It is interesting to remark here that as fluid moves to the separation points of reverse roll-coating, the viscosity of the fluid increases and the coating of the web is done after this position.

**Figure 8:**
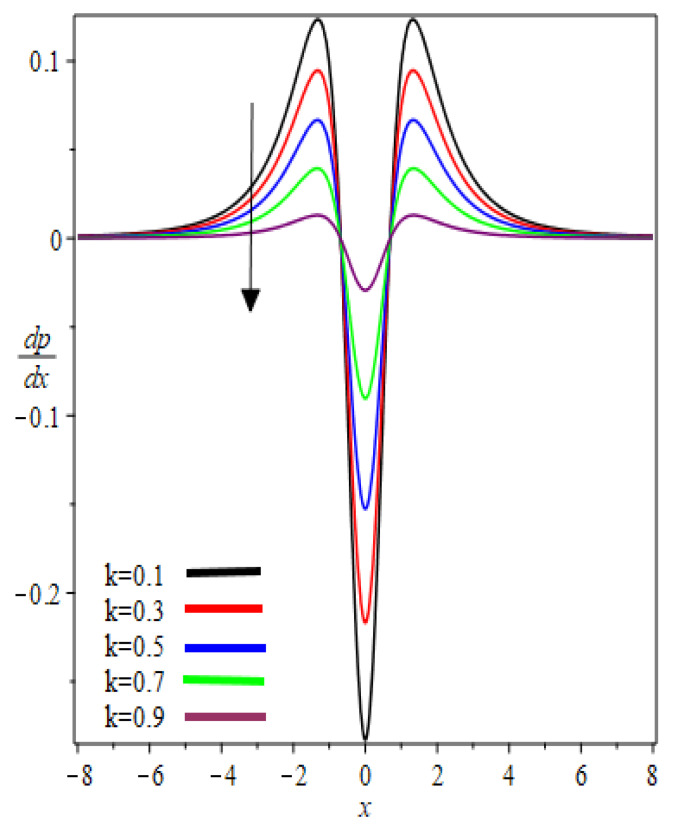
k Influence on pressure gradient.

**Figure 9:**
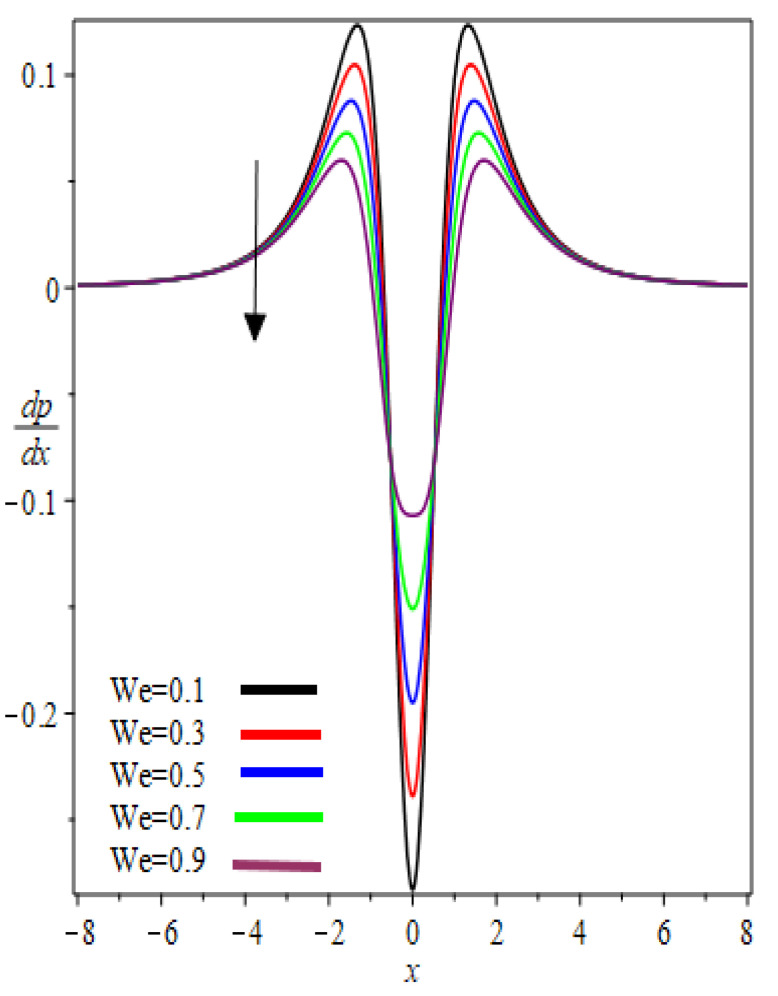
We Influence on pressure gradient.

The graphic results for pressure gradient $\frac{dp}{dx}$ against the axial coordinate x for the several involved parameters are presented in Figure 8 and Figure 9. From Figure 8 and Figure 9, it was observed that the symmetric profiles about the nip region x=0 are found. At nip region, the pressure gradient is negative and increases symmetrically, reaches the maximum value, then decreases exponentially and reaches zero at separation points. One can observe that for a particular value of k and We, the pressure gradient distribution increases. In addition, these material parameters have a significant effect on the pressure gradient at the nip region because the absolute value of the pressure gradient is maximum at this point. In Figure 8 and Figure 9 Newtonian results [13] are obtained as We→0.

**Figure 10:**
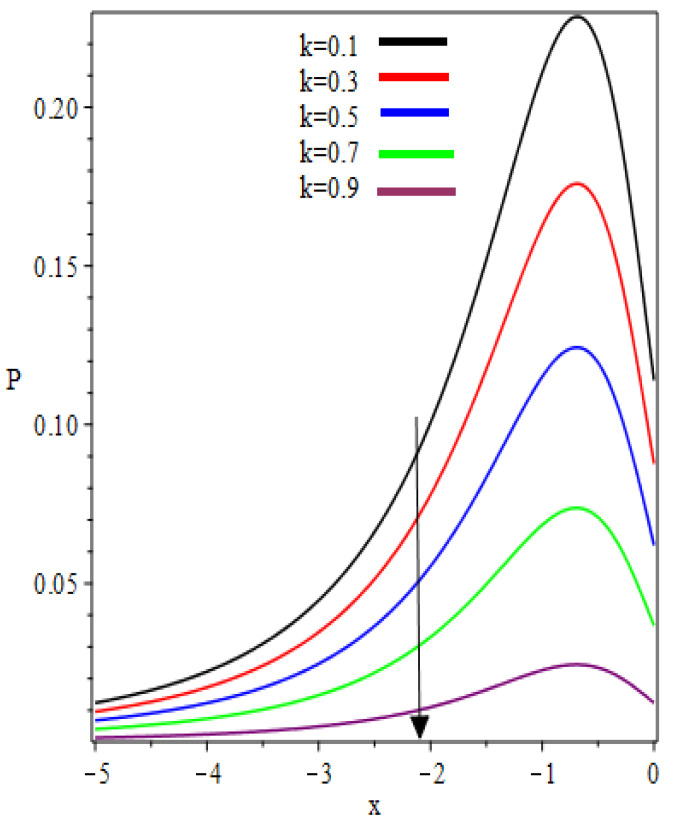
k Influence on the pressure distribution.

**Figure 11:**
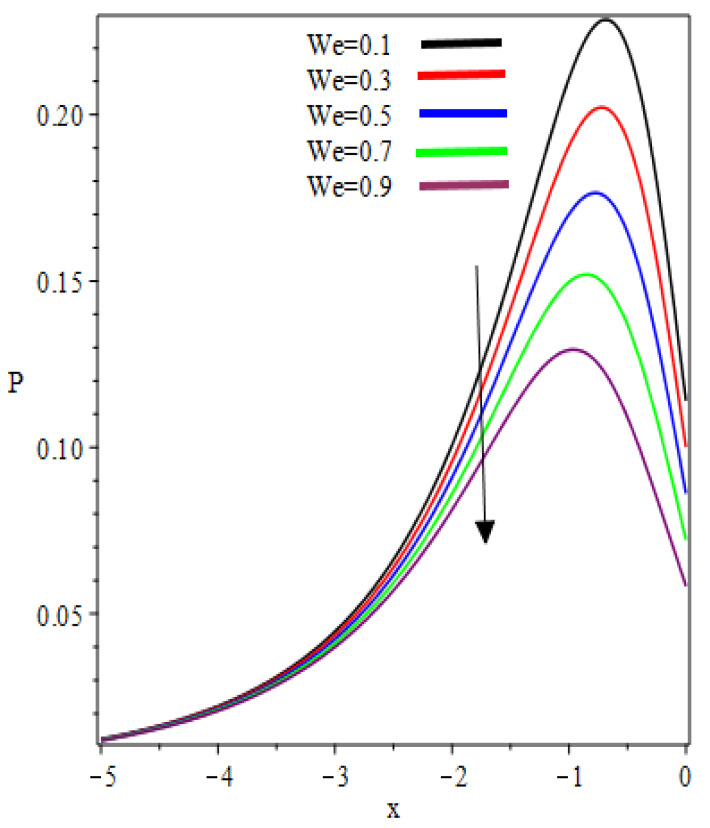
We Influence on the pressure distribution.

The graphic representation of pressure distribution for the increasing values of k and We is sketched in Figure 10 and Figure 11, respectively. From Figure 10, it is observed that when one starts moving toward the negative x−axis, the value of pressure increases and the maximum pressure occurs at the critical point x=−0.6; then the pressure starts decreasing. The concavity changes in the interval (−1.321,0) and (0,1.321) (not shown in the graph). Similar behaviors can be found in Figure 11.

**Figure 12:**
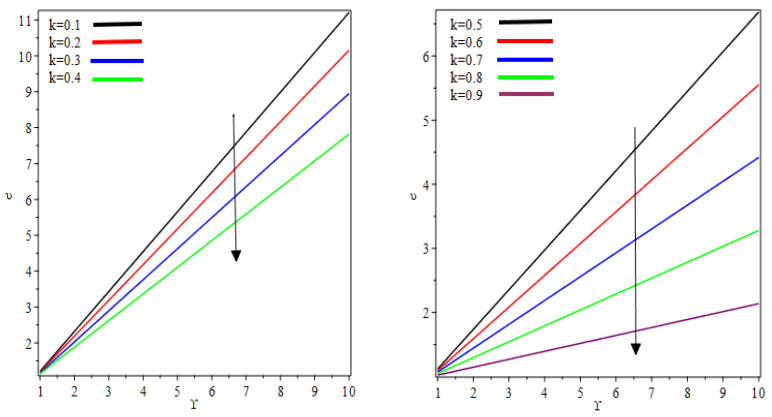
Thickness of coating versus ϒ for different valve of k.

The numeric results for flow rate, separation points, the thickness of the coating, separating force and power input are presented in Table 1 and Table 2 for the different valves of k and We. From Table 1, it is observed that the maximum thickness of the coating is 2.3216 for k=0.1. The coating thickness decreases with an increase in k. The minimum thickness of coating 1.1476 was found at k=0.9. The flow rate, separation points and thickness of coating are decreasing functions of k. The roll-separation force decreases, whereas the magnitude of power input increases with an increase in k. It can be seen from Table 2 that the thickness of the coating to the maximum can be as high as 3.2452 against the separation point 1.1436. The minimum thickness of the coating was observed as 2.3216. The flow rate, separation points and thickness of coating are increasing functions of We. It can be seen that the magnitude of roll-separation force and power input decreases by increasing the value We. We found that when We→0 all the results of Middleman [13] are retrieved. The graphic relation between the thickness of the coating and the ratio of half of the nip region to incoming fluid film against various values of velocities ratio k is presented in Figure 12. It can be seen that thickness of the coating is decreasing function of velocities ratio.

## 7. Conclusions

In this paper, we present the theoretical assessment of reverse roll-coating with the principle of lubrication approximation theory for an incompressible Williamson fluid. The analytical solutions for velocity profiles, pressure gradients and flow rates are obtained by using regular perturbation techniques. The separation points, thickness of the coating, separation force and power input are tabulated in numeric form. The key deductions of the present study are as follows:

➢ Flow velocity increases as We increases.
➢ Maximum velocity occurs at the roll surface of the reverse roll.
➢ Absolute pressure gradient is maximum at the nip point.
➢ Viscous forces are dominant over the elastic forces.
➢ Concavity of the pressure distribution changes in the interval (−1.321,1.321).
➢ The velocity ratio parameters and Weissenberg number play important roles in controlling the pressure gradient and pressure distribution.
➢ The Weissenberg number provides an economical mechanism to control the magnitude of separation force and power input.
➢ The Weissenberg number also plays fundamental role to have flow rate, separation points and coating thickness as per desirous.
➢ Pressure distribution, power input and the viscous forces play a significant role in coating thickness.
➢ As a fluid moves to the separation point of reverse roll-coating, the viscosity of the fluid increases and the coating of the web is done after this position.
➢ If We→0, the results of [13] are recovered.

## 8. Future Work

The effects of heat and the porous medium were not considered in this study. In the future, the effect of heat and porous media will be explored for more complicated rheological non-Newtonian models.

## Figures and Tables

**Table 1:** Influence of k on flow rate λ, separation points $x_{t}$ and thickness of coating υ, separation force F and power input $p_{w}$.

k	λ	$x_{t}$	$\upsilon =\frac{H_{f}}{H_{r}}$	F	$p_{w}$
0.1	0.5554	0.7243	2.3216	0.2262	−1.0113
0.2	0.4978	0.7242	2.1912	0.1999	−1.0566
0.3	0.4325	0.7241	2.0300	0.1742	−1.1013
0.4	0.3709	0.7239	1.8836	0.1485	−1.1454
05	0.3093	0.7238	1.7372	0.1231	−1.1891
0.6	0.2476	0.7236	1.5904	0.0981	−1.2321
0.7	0.1859	0.7234	1.4436	0.0728	−1.2751
0.8	0.1240	0.7230	1.2960	0.0483	−1.3169
0.9	0.0619	0.7218	1.1476	0.0244	−1.3575

**Table 2:** Influence of We on flow rate λ, separation points $x_{t}$ and thickness of coating υ, separation force F and power input $p_{w}$.

We	λ	$x_{t}$	$\upsilon =\frac{H_{f}}{H_{r}}$	F	$p_{w}$
0.1	0.5554	0.7243	2.3216	0.2262	−1.0113
0.2	0.5593	0.7767	2.4372	0.2128	−0.9696
0.3	0.5632	0.8291	2.5528	0.1994	−0.9172
0.4	0.5670	0.8815	2.6680	0.1859	−0.8320
0.5	0.5708	0.9339	2.7832	0.1722	−0.7966
0.6	0.5747	0.9863	2.8988	0.1583	−0.7235
0.7	0.5785	1.0387	3.0140	0.1442	−0.6428
0.8	0.5824	1.0912	3.1296	0.1298	−0.5546
0.9	0.5863	1.1436	3.2452	0.1149	−0.4588

## Abbreviations

$U_{r}$	Peripheral velocity of the reverse roll
$U_{f}$	Peripheral velocity of forwarding roll
R	Radius of the roll
$k=\frac{U_{r}}{U_{f}}$	Velocities ratio
$\bar{\tau }$	Extra stress tensor
ρ	Density
γ	Share rate
π	Second invariant strain tensor
We	Weissenberg number
$H_{0}$	Half the nip separation
$H_{r}$	Thickness of the coating on reverse roll
$H_{f}$	Thickness of the coating on the forwarding roll
$\upsilon =\frac{H_{f}}{H_{r}}$	Coating Thickness
$ϒ=\frac{H_{0}}{H_{r}}$	Ratio of the half of the nip region to the coating thickness on the reverse roll
λ	Dimensionless flow rate
